# Automatic day-2 intervention by a multidisciplinary antimicrobial stewardship-team leads to multiple positive effects

**DOI:** 10.3389/fmicb.2015.00546

**Published:** 2015-06-03

**Authors:** Jan-Willem H. Dik, Ron Hendrix, Jerome R. Lo-Ten-Foe, Kasper R. Wilting, Prashant N. Panday, Lisette E. van Gemert-Pijnen, Annemarie M. Leliveld, Job van der Palen, Alex W. Friedrich, Bhanu Sinha

**Affiliations:** ^1^Department of Medical Microbiology, University of Groningen, University Medical Center GroningenGroningen, Netherlands; ^2^Certe Laboratory for Infectious DiseasesGroningen, Netherlands; ^3^Department of Clinical Pharmacy and Pharmacology, University Medical Center GroningenGroningen, Netherlands; ^4^Department of Psychology, Health and Technology, University of TwenteEnschede, Netherlands; ^5^Department of Urology, University Medical Center GroningenGroningen, Netherlands; ^6^Department of Research Methodology, Measurement and Data Analysis, University of TwenteEnschede, Netherlands; ^7^Department of Epidemiology, Medisch Spectrum TwenteEnschede, Netherlands

**Keywords:** A-Teams, antimicrobial prescription, antimicrobial stewardship, antimicrobial resistance, intervention study

## Abstract

**Background:** Antimicrobial resistance rates are increasing. This is, among others, caused by incorrect or inappropriate use of antimicrobials. To target this, a multidisciplinary antimicrobial stewardship-team (A-Team) was implemented at the University Medical Center Groningen on a urology ward. Goal of this study is to evaluate the clinical effects of the case-audits done by this team, looking at length of stay (LOS) and antimicrobial use.

**Methods:** Automatic e-mail alerts were sent after 48 h of consecutive antimicrobial use triggering the case-audits, consisting of an A-Team member visiting the ward, discussing the patient’s therapy with the bed-side physician and together deciding on further treatment based on available diagnostics and guidelines. Clinical effects of the audits were evaluated through an Interrupted Time Series analysis and a retrospective historic cohort.

**Results:** A significant systemic reduction of antimicrobial consumption for all patients on the ward, both with and without case-audits was observed. Furthermore, LOS for patients with case-audits who were admitted primarily due to infections decreased to 6.20 days (95% CI: 5.59–6.81) compared to the historic cohort (7.57 days; 95% CI: 6.92–8.21; *p* = 0.012). Antimicrobial consumption decreased for these patients from 8.17 DDD/patient (95% CI: 7.10–9.24) to 5.93 DDD/patient (95% CI: 5.02–6.83; *p* = 0.008). For patients with severe underlying diseases (e.g., cancer) these outcome measures remained unchanged.

**Conclusion:** The evaluation showed a considerable positive impact. Antibiotic use of the whole ward was reduced, transcending the intervened patients. Furthermore, LOS and mean antimicrobial consumption for a subgroup was reduced, thereby improving patient care and potentially lowering resistance rates.

## Introduction

Antimicrobial resistance is a world-wide problem. Suboptimal prescription and subsequent inappropriate use of antimicrobials contributes to increasing development of resistance (Goossens, 2009; Tacconelli et al., 2009). The optimization of antimicrobial therapy in hospitalized patients is therefore an urgent global challenge (World Health Organization, 2012; Bartlett et al., 2013). Urology departments are even more vulnerable because of a high number of (high risk) gram-negative bacteria species encountered (Wagenlehner et al., 2013). Several aspects regarding antimicrobial therapy such as choice of drug (and spectrum), duration, mode of administration and dosage should be subject for improvement (Braykov et al., 2014). This is also true for countries with a relatively low antimicrobial prescription rate and low resistance rates, such as the Netherlands (de Kraker et al., 2013; European Centre for Disease Prevention, 2013). Therefore, Dutch government has made an antimicrobial stewardship program (ASP) with A-Teams mandatory for every hospital from January 2014 (SWAB, 2012).

Antimicrobial stewardship addresses many aspects of infection management, of which one is audit and feedback of the therapy (Davey et al., 2013). In recent years this has proven to improve on the appropriateness of antimicrobial therapy (Senn et al., 2004; Pulcini et al., 2008; Cisneros et al., 2014; Liew et al., 2015). At the University Medical Center Groningen (UMCG) in the Netherlands, this case-audit is combined with face-to-face consultation on day 2. The goal is to optimize and streamline the antimicrobial therapy as early as possible using microbiological diagnostics, thereby improving patient care. Face-to-face consultations are used explicitly to create an effective learning moment for prescribing physicians (Lo-Ten-Foe et al., 2014).

The aim of this study is evaluating this implemented A-Team on a urology ward in an academic hospital setting, focusing on two clinical outcome measures: antimicrobial use and length of stay (LOS). Because there are considerable risks of acquiring a nosocomial infection with each extra day of hospitalization (Rhame and Sudderth, 1981) and acquiring a catheter-related infection with each extra day of an intravenous line (Sevinç et al., 1999; Chopra et al., 2013), changes in LOS and/or antimicrobial administration can have a large impact on the quality of care and patient safety. The direct return on investment for this A-Team has already been extensively evaluated separately, using the same patient groups, and found to be positive (Dik et al., 2015a). The clinical outcome measures in this study have been evaluated through an interrupted time-series analysis as well as through a quasi-experimental set-up, thereby providing an extensive evaluation of a set of clinically relevant parameters.

## Materials and Methods

The study was performed at a 20-bed urology ward in a large 1339-bed academic hospital in the northern part of the Netherlands from June 16th 2013 to June 16th 2014. Inclusion of patients for the A-Team was done using a clinical rule program (Gaston, Medecs BV, Eindhoven, the Netherlands). The applied algorithm selected patients who received 48 h of selected antimicrobials (Supplementary Table S1). These were chosen based upon in-house evaluation of consumption at the ward and their respective risk for resistance development in general. In total 72% of the prescriptions for this ward was covered, including all drugs recommended in the applicable guidelines for the included patients. The clinical rule program automatically sends an email alert to the A-Team members (day 2), which contains the patient hospital ID, prescription details (e.g., administered antimicrobials, dosage, and start date [day 0]). It also includes clinical chemistry data (ALAT, ASAT, CRP, leucocytes, and creatinine). Microbiological diagnostic reports were collected manually. The hypothesis was that in the majority of the cases microbiologic diagnostic results will be (partly) available at day 2, and can thus be used during the case-audit. Furthermore, all A-Team members had access to all available microbiological data, including not yet finally authorized data (i.e., samples still being processed). Patients whose antimicrobial therapy started within 3 days after admission were included in the evaluation.

The A-Team is multi-disciplinary consisting of clinical microbiologists, infectious disease physicians, and hospital pharmacists. It reports to the hospital’s Antimicrobials Committee and Infection Prevention Committee, which have a mandate from the board of directors to implement and run the ASP. It is an integral part of one of the “leading coalitions” within the hospital to improve patient safety and quality of care. One A-Team member (clinical microbiologist or infectious disease physician) visited the ward after being triggered by the email alert and discussed antibiotic therapy with the bed-side physician(s) face-to-face. Consensus on the continuation of the treatment is one of the main goals. During the case-audit, the therapy was discussed and the available diagnostics were reviewed. Using the expertise and experience of both the A-Team member and the bed-side physician a decision on the continuation of the antimicrobial therapy was made. Decisions were always made based on local guidelines for urological infections, which in turn are based on national and European guidelines (Geerlings et al., 2013; Grabe et al., 2013). In the end, agreed-on interventions were scored. The chosen interventions were based upon a previous pilot in the same hospital (Supplementary Table S2; Lo-Ten-Foe et al., 2014).

Compliance was assessed at day 30. If the agreed-on intervention was followed by the appropriate action within 24 h, it was scored as compliant.

Evaluation of practice was the main goal of this study, focussing on a change in antimicrobial consumption and a reduction in LOS. Based upon the pilot study (Lo-Ten-Foe et al., 2014), a reduction of at least 1 day was our pre-determined goal. A financial evaluation of the same group of patients and using the same historic cohort was already performed (Dik et al., 2015a). Implementation of the A-Team is part of a local hospital-wide ASP, which is being developed keeping in mind recommendations done by the IDSA/SHEA and the ESGAP (Keuleyan and Gould, 2001; Dellit et al., 2007).

### Historic Control Cohort

For evaluation of the clinical effects, a frequency based historic control cohort was compiled. The control cohort consisted of patients who stayed at the same ward in a 30-months period prior to the intervention. Diagnosis Related Group (DRG) codes from the patients in the intervention group and the consumption of >48 h of the alert antimicrobials (Supplementary Table S1) were used to filter the control cohort. DRG codes were assigned to the patient after discharge for insurance purposes by a grouper, based upon scored procedures^1^. Patients’ antimicrobial consumption was measured in DDDs per 100 patient days, as stated by the WHO^2^.

The described case-audit was normal every day care implemented within the hospital and approved by the Antimicrobials Committee, following national guidelines. This study was of an observational nature, evaluating the newly implemented procedures. Data was collected retrospectively from the hospital’s data warehouse; it was anonymous, partly aggregated and did not contain any directly or indirectly identifiable personal details. Following Dutch legislation and guidelines of the local ethics committee, formal ethical approval was therefore not required^3^.

### Subgroup Analysis

After explorative analysis of the data two subgroups were compiled to correct for the modifying effect of the patients’ indication. The two groups were stratified by DRG codes. The first group (Group 1) consisted of patients without severe underlying diseases and whose infection was the most likely major problem and the main driver for LOS. The second group (Group 2) consisted mainly of oncology and transplantation patients. Here the underlying problem (e.g., cancer) was the most likely driver for LOS, rather than the infection.

### Statistical Analysis

For antibiotic consumption of the total ward, including patients without intervention(s), an interrupted time-series analysis was performed. For subgroup analysis, unpaired *t*-tests, chi square tests and Kaplan–Meier survival plots with a log-rank test were applied, as appropriate. Significance threshold was *p* < 0.05. For subgroup analyses, a threshold of *p* < 0.025 was set in order to account for possible family wise error rates. Analysis was done with IBM SPSS Statistics 20 (IBM, Armonk, NY, USA) after 1 year.

## Results

### Consulted Patients

During the 1-year study period, 1298 patients were admitted to the urology ward. 850 received at least one dose of antimicrobials. 114 alert patients were included in this study (61% male; mean age 62 years male, 50 years female; **Table 1**). They received a total of 126 case-audits (including 12 follow-up consults), resulting in 166 interventions. Consensus was reached in 97.6% of the cases (*n* = 123) and the compliance (i.e., action within 24 h) was 92.1% (*n* = 116). Case-audits took on average between 10 and 15 min, including administration time.

**Table 1:** Patient baseline characteristics.

	Intervention group	Control group	*p*-value
Total group	*N* = 114	*N* = 357	
Male	61%	69%	0.11^a^
Mean age	57.51 (±2.95) years	61.52 (±1.72) years	0.01^b,c^
Group 1	*N* = 70 (61%)	*N* = 209 (59%)	
Male	51%	60%	0.11^a^
Mean age	55.25 (±3.71) years	59.35 (±2.25) years	0.046^b^
Group 2	*N* = 44 (39%)	*N* = 148 (41%)	
Male	75%	84%	0.71^a^
Mean age	61.12 (±4.79) years	64.57 (± 2.62) years	0.14^b^

Included patients and the control group patients’ characteristics and their respective ox*p*-values. 95% Confidence intervals are shown in brackets, when applicable. Characteristics are given for the total group, as well as for the two subgroups. Group 1 consisted mainly of patients without severe underlying diseases, and Group 2 consisted of patients with severe underlying diseases. ^a^Chi square test; ^b^Mann–Whitney *U-T*est; ^c^Total group was not used further in calculations. The difference in age and sex showed no significant influence on the outcome measures.

### Results of Microbiological Diagnostics were Mostly Available on Day 2

In 86.0% (*n* = 98) of the alert patients microbiological diagnostics had been initiated, in 50.0% (*n* = 57) this was done on day 0 or 24 h prior to starting antimicrobials. At the first case-audit (day 2) results were (partly) available (gram staining, incomplete culture data) in 72.8% (*n* = 83) of the cases.

### A Large Majority of the Consulted Patients Received Interventions

**FIGURE 1:**
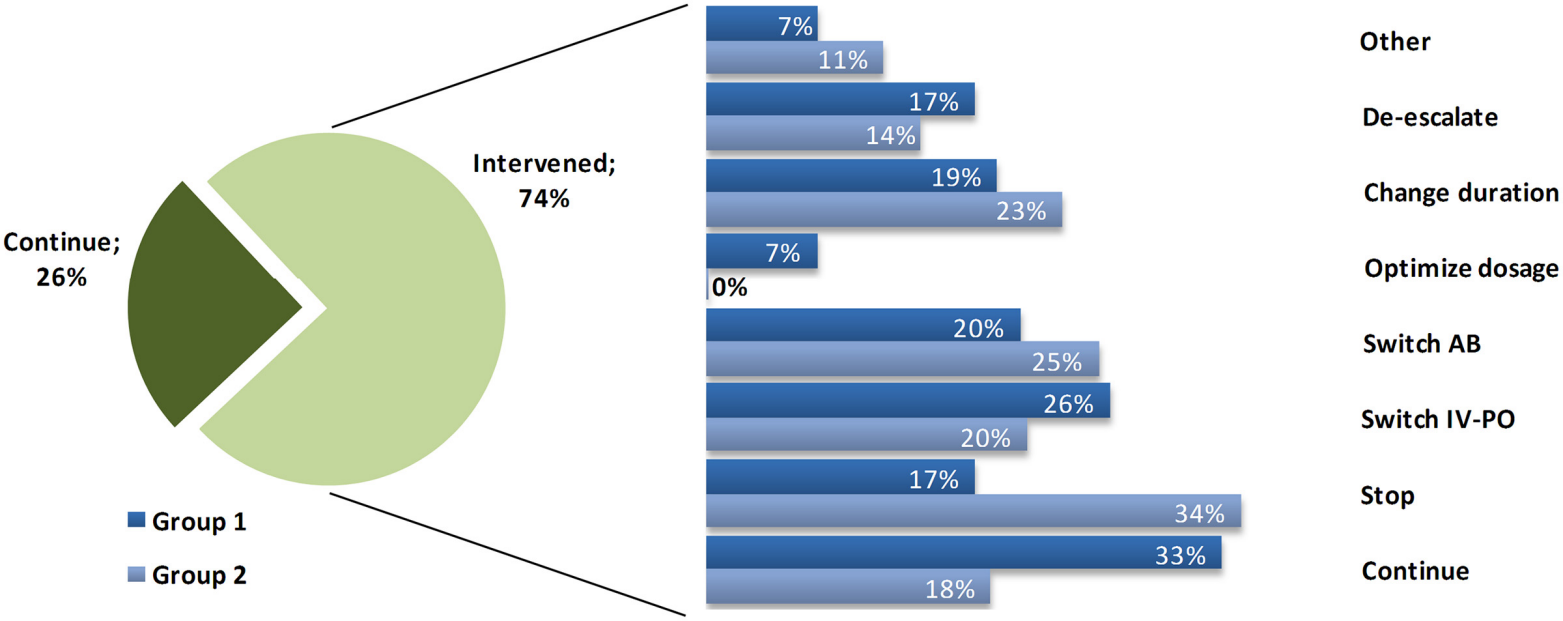
**Interventions performed**. Distribution of the interventions performed for alert patients, subdivided into the two Groups. Percentages of interventions refer to the total number done within the 75% of intervened patients, where one patient can receive multiple interventions.

Of the patients who were consulted, there was an alteration of the therapy (any intervention besides ‘continue’ at the first case-audit) in 74.7% of the patients. In 23.7% (*n* = 27; 16.3% of total interventions) treatment was stopped. A switch to oral treatment was performed in 23.7% (*n* = 27; 16.3% of total interventions). 21.9% (*n* = 25; 15.1% of total interventions) received a different antimicrobial, dosage was optimized in 4.4% (*n* = 5; 3.0% of total interventions) and treatment de-escalated in 15.8% (*n* = 18; 10.8% of total interventions). For 8.8% (*n* = 10; 6.0% of total interventions) there another intervention (e.g., add an antimicrobial, perform extra diagnostics) was performed (see **Figure 1** for the stratification of interventions per subgroup).

### Prescribing Trends of the Whole Ward Changed after Implementation

**FIGURE 2:**
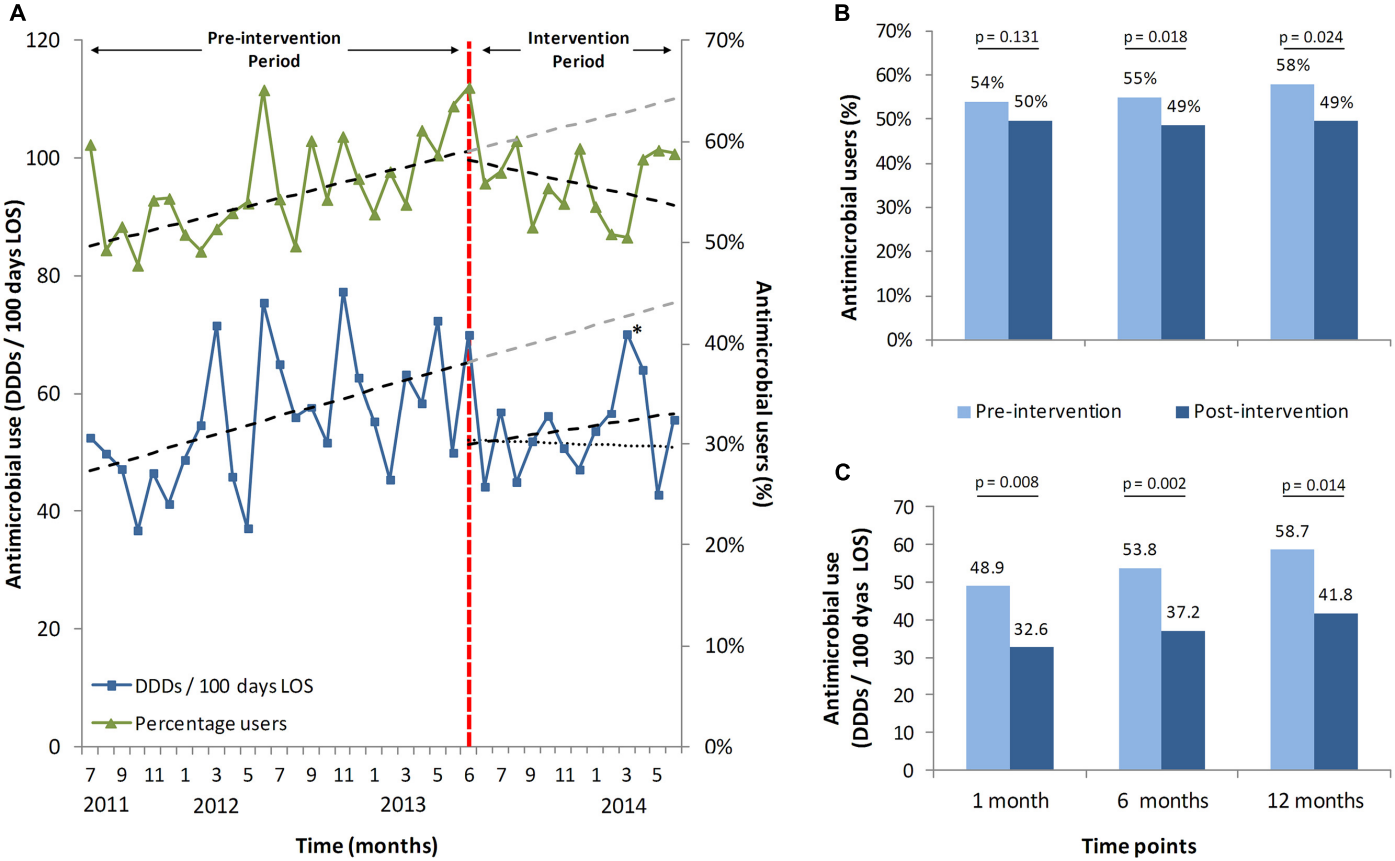
**Antimicrobial stewardship-team (A-Team) effects on the whole ward. (A)** trends of percentages of all patients on the ward receiving antibiotics with and without intervention(s) and respective DDDs per 100 patient days. Shown are 2 years before the intervention started (June 2013), until June 2014, including trend lines and predicted trend lines. A second dotted trend line for the mean DDDs depicts the trend without the outlier patient from April 2014 (^∗^). **(B)** predicted and measured percentages of users at three different time points with their respective *p*-values, calculated with an interrupted time series analysis. **(C)** predicted and measured consumption with their respective *p*-values, on the same three time points with the same interrupted time series analysis. (^∗^) The peak in the month April is caused by a single patient who received correct but extensive small spectrum oral antibiotic treatment for a deep-seated, complicated infection.

Most notably, the positive effect transcended the target group on this ward. The trend of antimicrobial consumption of all patients admitted to the ward (17.3% intervened and 82.7% not intervened) changed after start of the intervention. Using an interrupted time-series analysis there was an observed drop of 25.0% after 1 month (*p* = 0.012), 23.6% at 6 months (*p* = 0.007), and 22.4% at 12 months (*p* = 0.047), compared to expected usage, based upon the extrapolated pre-intervention data (**Figures 2A,C**).

The mean percentage of antimicrobial recipients per month in relation to the total number of patients dropped by 7.3% at 1 month (*p* = 0.131), 10.4% at 6 months (*p* = 0.018) and 12.8% at 12 months (*p* = 0.024), compared to the expected percentage of recipients (**Figures 2A,B**).

### Length of Stay was Significantly Reduced for Group 1 Patients

**FIGURE 3:**
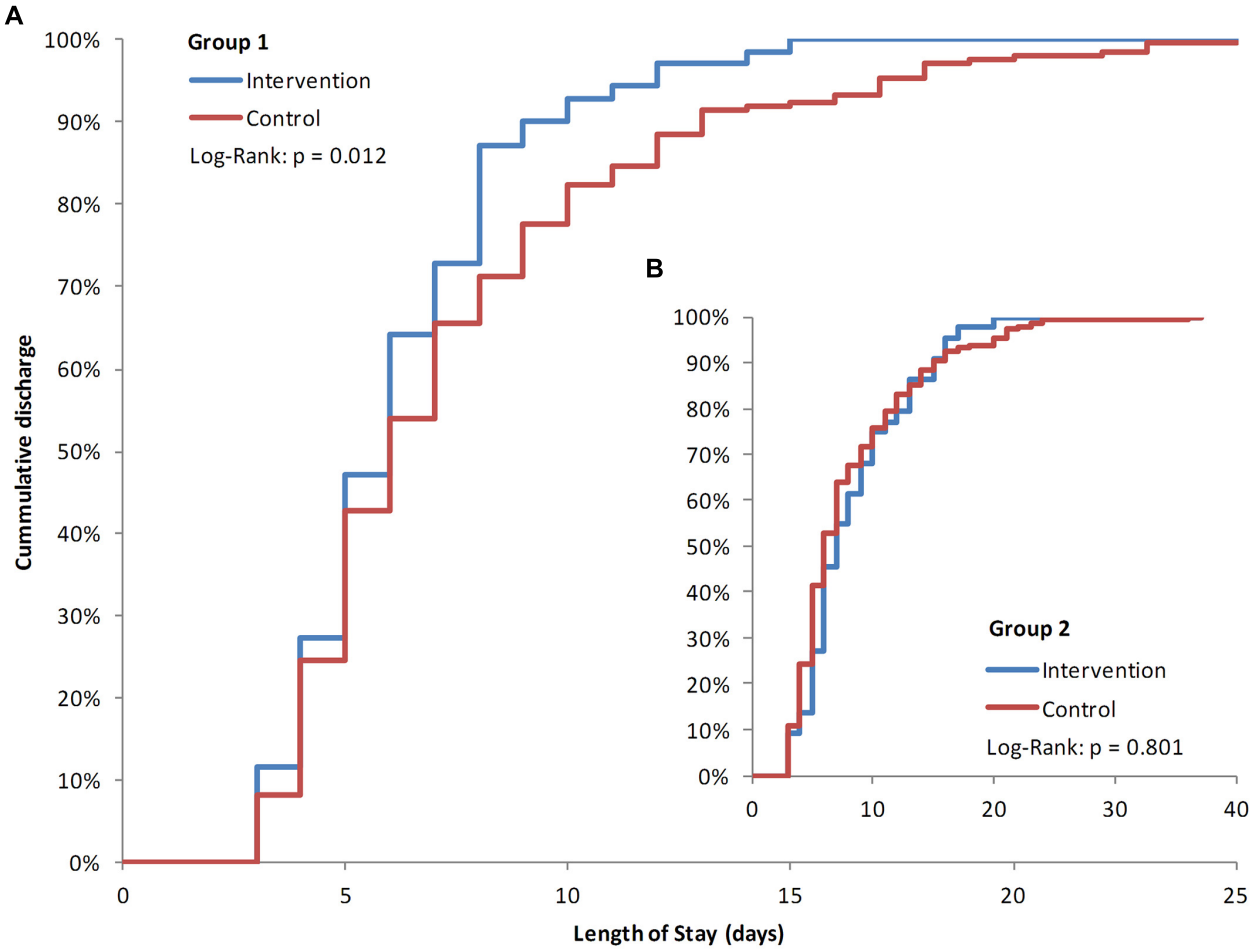
**Kaplan–Meier plots of length of stay (LOS)**. Percentages of patients’ days of discharge. Group 1 intervention patients compared to the historic cohort group 1 **(A)** with Group two patients as insert **(B)**. Significance was tested with a Log–Rank test (Mantel–Cox).

Length of stay was evaluated for the two subset groups to take into account the modifying effect of the patients’ indication. Group 1, without severe underlying diseases, showed an average LOS reduction of 1.46 days compared to the control group (6.20 days; 95% CI: 5.59–6.81 vs. 7.57 days; 95% CI: 6.92–8.21; *p* = 0.012; **Figure 3A**). LOS for patients of Group 2, with severe underlying disease had a minor but non-significant increase compared to the control group (8.36 days; 95% CI: 7.10–9.62; vs. 8.10 days; 95% CI: 7.24–8.97; *p* = 0.801; **Figure 3B**). Patients’ LOS remained the same for patients who stayed at the department and did not receive an intervention (3.95 days; 95% CI: 3.75–4.16) compared to 1 year earlier (3.96 days; 95% CI: 3.71–4.21; *p* = 0.581).

### Antimicrobial Consumption was Lower for Group 1 Patients

Overall antimicrobial consumption dropped by 2.24 DDD per patient for Group 1 (*p* = 0.008). There was a trend to lower intravenous administration (67%, compared to 69% in the control cohort; *p* = 0.099). For Group 2 there was a non-significant drop of 0.91 DDD per patient (*p* = 0.712; **Table 2**).

**Table 2:** Effects of the Antimicrobial Stewardship-Team (A-Team) interventions on antibiotic use (DDD per patient).

		Intervention group	Control group	Difference	*p*-value
Group 1		*N* = 70	*N* = 209		
	Overall DDDs	5.93 (±0.90)	8.17 (±1.07)	-27.5%	0.008
	PO DDDs	1.95 (±0.52)	2.51 (±0.44)	-22.3%	0.849
	IV DDDs	3.97 (±0.93)	5.66 (1.05)	-29.8%	0.099
Group 2		*N* = 44	*N* = 148		
	Overall	7.21 (±1.47)	8.13 (±1.11)	-11.2%	0.712
	PO	2.98 (±0.92)	2.81 (±0.59)	5.9%	0.931
	IV	4.20 (±1.36)	5.31 (±0.95)	-20.3%	0.738

Antibiotic consumption compared between the intervention group and the historic control cohort for Group 1 and Group 2. Consumption is presented as mean DDDs per patient, the difference between the intervention and control in percentages and 95% CI in brackets. All tests were unpaired, two-tailed ox*t*-tests performed on log-transformed data.

## Discussion

The main goal of this study was to evaluate an already implemented A-Team and the effects of its case-audits, by looking both at LOS and DDDs. These parameters were taken as main outcome measures, because they are known to have a major impact on the quality of care, and they are affected by an ASP (Davey et al., 2013). The A-Team reviewed antimicrobial therapy on day 2, thus, making optimal use of available (microbiological) diagnostics. By means of the automatic alert (which can be modified for specific groups of patients, departments, and specific antimicrobials) face-to-face case-audit on the ward was encouraged and facilitated by providing an easy overview of relevant patient information. Objective of the case-audit was to reach a consensus-based agreement between the A-Team member and the physician at the ward, using (local) guidelines, available diagnostics and the expertise and experience of both physicians. This should optimize antimicrobial treatment. The case-audit focused on the improvement of patient care through relatively easy to achieve improvements after only 48 h of therapy, such as an early IV-PO switch, and stopping therapy when there was no longer an indication. Furthermore, the bed-side physicians anticipated the A-Team visits. These provided an extra opportunity for questions about appropriateness of therapy and requesting additional consultations for further patients on the ward. This resulted in 19 additional patients discussed during the evaluation period. These patients had not triggered an alert, for example because therapy was still less than 48 h or because therapy had not even been started. Twelve patients received more than one case-audit. These follow up audits were often deemed necessary because culture results were not completely available.

Very unexpectedly, and unlike previously published in other studies, we found a considerable significant additional positive effect on the antimicrobial consumption of the whole ward. With consultation of 17.3% of the patients at the ward during the evaluation period, we saw a broad effect on all patients, including those without any consultations or intervention(s). The drop in the rate of patients receiving antimicrobials and the drop in DDDs per patient has been very likely due to the continuing educational effect of the consensus-based case-audit where face-to-face information exchange is taking place. Although antimicrobial consumption of the whole ward would be directly affected by interventions, the percentage of recipients should not change. This strengthens the conclusion that the A-Team presence at the ward had an effect transcending the group of intervened patients. The knowledge that antimicrobial use is monitored and evaluated will most likely contribute to a higher awareness by the prescribing physicians, thereby possibly creating a kind of Hawthorne effect (Mangione-Smith et al., 2002). Going to wards to discuss patients requires investment of staff time. However, it should be noted that for this ward, on average only 10–15 min were spent per case-audit by an A-Team member, including administration. It was not the goal to discuss every patient with antimicrobial therapy. A large majority of patients received prophylactic therapy of which the duration should be less than 48 h (often just a single shot) and where an intervention would not achieve a lot of effect. The quality of economic analyses of ASPs is often sub optimal, due to insufficient outcome measures and performed methods (Dik et al., 2015b). Therefore, a more extensive and thorough economical analysis has been also undertaken for the same patients and using the same historic cohort. Mainly due to the decrease in LOS for the group of patients primarily admitted due to an infection, the implementation of the A-Team had a positive direct return on investment (Dik et al., 2015a).

Automatic email alerts can be easily adjusted to the specific needs of a hospital or ward, depending on local challenges and required goals (e.g., IV-PO switch, review of only restricted antimicrobials, only one ward or the whole hospital, children, or adults, and at a timeframe of choice), making it a relatively simple method to keep track of patients receiving (selected) antimicrobials. We estimated that implementation of this specific program for the whole hospital would require 2–3 fte A-Team specialists.

The large number of performed interventions shows that implementing an A-Team was highly relevant. Indeed, frequent non-optimal antimicrobial treatment of urology patients has been shown earlier (Hecker et al., 2014). Antimicrobial treatment can be improved also in other patient populations (Braykov et al., 2014). In line with our results, an audit and feedback of therapy after 48 h has been recently shown to be fruitful in a hospital-wide setting (Liew et al., 2015). However, this study did not show a reduced LOS as a benefit. Although difficult to compare due to the different setting, the higher availability of microbiological diagnostics in our patients might account for the differences in LOS. Furthermore, patient it is important to take the characteristics of the patient group into account.

Here, we observed that the interventions resulted in a reduced average LOS for a subgroup of patients and a global drop in antimicrobial DDDs. This finding underscores that a substantial proportion of patients can be switched earlier to oral administration or stopped completely, an easily achievable target, the “low hanging fruits” (Goff et al., 2012). The IV-PO switches also explain why oral administration did not change significantly. However, the effects on oral DDDs might even be larger, because 23% of the IV to PO switch patients was sent home directly after the switch without getting inpatient oral therapy. Consequently, they did not count for the calculation of mean DDDs for PO treatment.

By lowering LOS and DDDs the risks for hospital acquired infections, catheter-related infections and resistance development should also be lower, thereby improving patient care and safety (Rhame and Sudderth, 1981; Sevinç et al., 1999; Chopra et al., 2013). However, the current time-frame of the study is too short to measure these effects and they are thus to be investigated in the coming years. Other outcome measures, notably duration of therapy in days and re-admissions rates were not significantly altered (data not shown). If an ASP were implemented for more complex patients (e.g., on an ICU), it would also be important to take morbidity and/or complications, and mortality into account, because these outcome measures can be expected to affect mainly more complex patients.

It is important to note that LOS may not be taken as a universal outcome measure or quality indicator for an ASP. As we conclusively show, a day 2 bundle will not lower the LOS for all patients. Especially in an academic hospital there are many referral patients with severe oncology or transplant surgery indications. If these patients subsequently develop an infection, the driver for the LOS and/or their antimicrobial use is most likely the underlying disease and a decrease in outcome measures such as LOS can thus not be expected. This fact could also explain ambiguous results on patients’ LOS seen in the Cochrane Review (Davey et al., 2013). Consequently, this point should be taken into account for the design and analyses of future ASP studies. Finally, it should be noted that the success of the program is not determined by a decrease in LOS or DDDs. The main goal was to improve the quality of care by adjusting therapy as quickly as possible according to the diagnostic results. This had an effect on LOS and DDDs on a subgroup of patients. Of note, this does not imply that the intervention failed in patients of group 2. Rather, this suggests that outcome measures to monitor success of an ASP have to be chosen wisely. Possible (in)direct effects extend much further, such as a lower risk of resistance and better quality of care for the patients due to a more optimal antimicrobial treatment. However, these outcome measures are difficult to measure objectively, especially on the short term in a retrospective set-up.

Our study has some limitations. Effects of the A-Team were evaluated for a urology ward in an academic setting. To investigate effects in different settings reliably, further studies are needed. Possible confounders with an effect on the LOS due to the quasi-experimental set-up and the chosen cohort might have been present, rendering ASP studies complicated to perform (Marwick and Nathwani, 2014). This study evaluated the effects of an already implemented intervention. Performing a (randomized) controlled trial was therefore neither an option nor a goal. Analyses were done within subgroups, to exclude modifying effects of the underlying disease. Without such a sub analysis, effects would be averaged out and lost. During the study period no other additional measures were performed to influence antimicrobial prescriptions or reduce LOS (i.e., changes in formularia, additional education or changes in restriction of antimicrobials). For antimicrobial consumption seasonal effects were ruled out by looking back 2 years. The distribution of age and sex was not optimal, but no correlation or effect was found on LOS or antibiotic use. The fact that the LOS of Group 2 did not change significantly provides an extra internal negative control. Unfortunately, not all information was available for evaluation (possible co-morbidities were not available as objective, measurable data).

We conclude that ASP interventions should be further encouraged. Inappropriate use of antimicrobials contributes to higher resistance rates, making infections even more difficult to treat (Burke and Yeo, 2004). Recently a Dutch multicenter study showed that appropriateness of antimicrobial therapy in UTI patients has a positive impact on the LOS (Spoorenberg et al., 2014), supporting our findings that the A-Team interventions successfully optimized therapy thereby reducing the LOS. Even though A-Teams cost money to implement, the reduction in LOS for some patients provides enough benefits to provide a positive return on investment (Dik et al., 2015a). Pro-active collaboration between the treating medical specialty, medical microbiology, infectious diseases, and pharmacy departments via a day-2 evaluation of antimicrobial therapies could be used to provide benefit for patients in other hospitals, as well.

## Author Contributions

JD, RH, JL, AF, and BS conceived the study; JD, KW, JL, and PN were involved in the execution of the study, JD and JP performed the analyses; JD, RH, LG, AF, and BS interpreted the data; JD, JL, and BS wrote the manuscript, and all authors critically read the manuscript, revised it and approved the final version.

## Conflict of Interest Statement

Jan-Willem H. Dik, Ron Hendrix, Jerome R. Lo-Ten-Foe, Kasper R. Wilting, Prashant N. Panday, Lisette E. van Gemert-Pijnen, Annemarie M. Leliveld, Job van der Palen, and AlexW. Friedrich all declared that they have no potential conflicts of interests. Bhanu Sinha has received a travel grant co-funded by Pfizer/Wyeth, and worked on projects in cooperation with Pathogenica Life Technologies, and Copan. The research was conducted in the absence of any commercial or financial relationships that could be construed as a potential conflict of interest.

## Footnotes

1 http://www.dbconderhoud.nl/

2 http://www.whocc.no/atc_ddd_index

3 http://www.ccmo.nl/en
