# Supplementary material for: Automatic day-2 intervention by a multidisciplinary antimicrobial stewardship-team leads to multiple positive effects
Source: Front Microbiol. 2015 Jun 3;6:546. doi: 10.3389/fmicb.2015.00546 (PMC4452884; doi:10.3389/fmicb.2015.00546)
Supplement: Supplementary file 1 [file Table_1.DOCX]

***Supplementary Material***

**Automatic day-2 intervention by a multidisciplinary Antimicrobial Stewardship-Team leads to multiple positive effects**

Jan-Willem H. Dik^1^, Ron Hendrix^1,2^, Jerome R. Lo-Ten-Foe^1^, Kasper R. Wilting^1^, Prashant Nannan Panday^3^, Lisette E. van Gemert-Pijnen^4^, Annemarie M. Leliveld^5^, Job van der Palen^6,7^, Alex W. Friedrich^1*^, Bhanu Sinha^1^

^1^Department of Medical Microbiology, University of Groningen, University Medical Center Groningen, Groningen, the Netherlands; ^2^Certe Laboratory for Infectious Diseases, Groningen, the Netherlands; ^3^Department of Clinical Pharmacy and Pharmacology, University Medical Center Groningen, Groningen, the Netherlands; ^4^Department of Psychology, Health and Technology, University of Twente, Enschede, the Netherlands; ^5^Department of Urology, University Medical Center Groningen, Groningen, the Netherlands; ^6^Department of Research Methodology, Measurement and Data Analysis, University of Twente, Enschede, the Netherlands; ^7^Department of Epidemiology, Medisch Spectrum Twente, Enschede, the Netherlands

**^*^Corresponding author:**

AWF Friedrich

Department of Medical Microbiology (HPC EB80)

University Medical Center Groningen

Hanzeplein 1

9713 GZ, Groningen, the Netherlands

Phone: +31-50-3613480

Fax: +31-50-3619105

Email: [alex.friedrich@umcg.nl](mailto:alex.friedrich@umcg.nl)

| **Supplemental Table S1: Alert antibiotics.**  The antibiotics included in the clinical rule with their respective ATC code and the percentage of the total use of the department before the implementation of the A-Team. | | | |
| --- | --- | --- | --- |
| **Group** | **Type/Name** | **ATC code** | **Consumption before intervention (%)** |
| Penicillins | Flucloxacillin | J01CF05 | 1.02 |
|  | Amoxicillin/Clavulanic acid | J01CR02 | 12.47 |
|  | Piperacillin/Tazobactam | J01CR05 | 2.46 |
| Cefalosporins | Cefuroxime | J01DC02 | 17.63 |
|  | Ceftriaxone | J01DD04 | 2.04 |
| Carbapenems | Meropenem | J01DH02 | 2.22 |
| Lincosamides | Clindamcyin | J01FF01 | 1.56 |
| Aminoglycosides | Tobramycin | J01GB01 | 4.98 |
| Fluoroquinolones | Ciprofloxacin | J01MA02 | 26.20 |
| Glycopeptides | Vancomycin | J01XA01 | 0.6 |
|  | Teicoplanin | J01XA02 | 0.78 |
|  | | | |

| **Supplemental Table S2: ASP Interventions.** The interventions done at the consulted patients and their respective definition. Interventions always regard the patients’ antibiotic therapy and not possible other medication. | |
| --- | --- |
| **Intervention** | **Definition** |
| Continue | No change in therapy, continue as is |
| Stop | Discontinue the therapy |
| Switch IV-PO | Switch the therapy from intravenous to oral^a^ |
| Switch AB | Switch an antibiotic |
| Optimize dosage | Change the dosage and/or dosing interval |
| Change duration | Change the duration or set a duration |
| De-escalate | Change from broad spectrum to a small spectrum antibiotic |
| Other | An intervention other than mentioned above^b^ |
| a) Switch to PO based upon national and thereupon based local guidelines (Sevinç et al., 1999; Handako et al., 2004) | |
| b) e.g. switch to IV; take cultures; add an antibiotic | |

**References supplemental material**

1. Handoko, K.B., van Asselt, G.J., and Overdiek, J.W. (2004). [Preventing prolonged antibiotic therapy by active implementation of switch guidelines]. *Ned. Tijdschr. Geneeskd.* 148, 222-226.

2. Sevinç F, Prins JM, Koopmans RP, Langendijk PNJ, Dankert J, and Speelman P. (1999). [Early change from intravenous to oral antibiotics: 'switch therapy']. *Ned. Tijdschr. Geneeskd.* 143, 2364-2365.
